# Influence of age and cognitive demand on motor decision making under uncertainty: a study on goal directed reaching movements

**DOI:** 10.1038/s41598-024-59415-7

**Published:** 2024-04-20

**Authors:** Melanie Krüger, Rohan Puri, Jeffery J. Summers, Mark R. Hinder

**Affiliations:** 1 Institute of Sports Science, Faculty of Humanities, Leibniz University Hannover, Am Moritzwinkel 6, 30167 Hannover, Germany; 2 Sensorimotor Neuroscience and Ageing Research Laboratory, School of Psychological Sciences, College of Health and Medicine, University of Tasmania, Hobart, Australia

**Keywords:** Human behaviour, Cognitive ageing

## Abstract

In everyday life, we constantly make decisions about actions to be performed subsequently. Research on motor decision making has provided empirical evidence for an influence of decision uncertainty on movement execution in young adults. Further, decision uncertainty was suggested to be increased in older adults due to limited cognitive resources for the integration of information and the prediction of the decision outcomes. However, the influence of cognitive aging on decision uncertainty during motor decision making and movement execution has not been investigated, yet. Thus, in the current study, we presented young and older adults with a motor decision making task, in which participants had to decide on pointing towards one out of five potential targets under varying cognitive demands. Statistical analyses revealed stronger decreases in correctly deciding upon the pointing target, i.e. task performance, from low to higher cognitive demand in older as compared to young adults. Decision confidence also decreased more strongly in older adults with increasing cognitive demand, however, only when collapsing across correct and incorrect decision trials, but not when considering correct decision trials, only. Further, older adults executed reaching movements with longer reaction times and increased path length, though the latter, again, not when considering correct decision trials, only. Last, reaction time and variability in movement execution were both affected by cognitive demand. The outcomes of this study provide a differentiated picture of the distinct and joint effects of aging and cognitive demand during motor decision making.

## Introduction

In everyday life, we constantly make decisions, with some of them having long-term consequences, such as deciding on financial investments in pension funds, while others may have more immediate consequences, such as deciding on a particular meal for lunch or which mug to grasp from a shelf. Thus, being able to decide between multiple choice options and to successfully execute the decision outcome represents a fundamental ability of everyday life. Consequently, decision making research has a long tradition in psychology as well as other fields of research (for a recent review on the integration of central theories which evolved from different disciplines, see^1^).

Importantly, decisions are often performed under uncertainty, which may arise from different origins^2^: First, *incomplete information*, where individuals have to perform decisions between two or multiple options, but do not have all the necessary information to decide on the “better” option. This may be the case when, for example, deciding upon whether to take a private car or public transport to arrive on time for a meeting, without knowing about the traffic conditions ahead. These circumstances are often referred to as decision making under *risk* or under *ambiguity*, depending on the nature of missing information (for a differentiation between the two, see^3^). Second, decision uncertainty may arise from *undifferentiated alternatives*, where individuals have all necessary information but two or more choice options are equally good. This may be the case when an individual can choose between two different flavors of ice cream, without having a preference for either one of the two. Last, decision uncertainty may result from *inadequate understanding*, where individuals have all necessary information to decide for the “better” of two or more choice options, but, due to individual (cognitive) resources or internal states^4,5^, are not able to adequately interpret this information. This may be the case in the context of e.g., learning processes or when not paying attention to relevant information. Thus, individual cognitive abilities seem to be of relevance for the decision process and its outcome, which leads to the question of how developmental changes of cognitive abilities in later life affect decision making.

Research interest into this direction has led to a breadth of experimental paradigms and methodological approaches used to study decision making under uncertainty across the lifespan (for a discussion on different theoretical and methodological perspectives, see^3^), targeting different domains and analytical levels (see e.g. domain^6^, neurochemical level^7^, neural level^8^). In this context, cognitive aging seems to be centrally affecting the perception of decision uncertainty and the prediction of decision outcomes, with age-related changes in prefrontal cortical activity reflecting changes in information integration relevant for the processing of potential gains and losses^8^. In this context, empirical evidence suggests decreased error awareness in decision making with increasing age^9^. Importantly, error awareness is assumed to play a central role in the “decision-making circuity”^10,11^, e.g. with regard to error detection and monitoring, which seem to share neurophysiological mechanisms with decision confidence judgement^12–14^. Further, it has been suggested that age-related limitations in available cognitive resources make the prospection of decision outcomes more challenging and less accurate, resulting in increased decision uncertainty in older adults^3^. One potential coping strategy for the age-related change in cognitive resources seems to be the reference to simpler, thus, less cognitively demanding decision strategies^15^, while another strategy might be decreased risk-taking^6,16–18^. These age-related changes in decision making under uncertainty might come with meaningful consequences for the execution of the decision outcome.

Decision outcomes are often executed in the form of goal-directed movements e.g., when deciding upon which mug to grasp from the shelf, as mentioned before, or when deciding upon which button to press on a ticket vending machine. Research interest in the processes underlying decision making on motor actions has significantly increased in recent years, see for example^19–22^. Fundamentally, motor decision making can be understood as the process of action plan selection in the presence of multiple competing, potential actions^23,24^, and is composed of an interplay of the perception of action opportunities, as well as the processes of action selection, planning, and execution. Thus, typical paradigms in perceptual decision making, where simple motor tasks are the mean to report the decision^25,26^, could in principle be also understood as one particular form of motor decision making, namely one in which different motor actions correspond to different choice options (one-to-one mapping), e.g. when pressing the left or right button to select either the left or right target. Beyond that, motor decision making comprises further forms of decision-action mappings, e.g. when selecting one out of different possible movement paths to reach the same target, and relates to various differently complex motor actions, e.g. pointing and reaching in three-dimensional space or walking^22,23^. Recent theories on motor decision making suggest that the processes involved in motor decision making run in parallel to account for and to be able to flexibly adapt to the dynamics and uncertainties in environmental conditions^19,20,27^. The behavioral implications of these theoretical assumptions have been widely tested using goal-directed pointing movements^28–30^. In these studies, in which decision uncertainty was induced by providing only incomplete information about the final pointing target until after movement onset (“go-before-you-know” set-up^29^), fingertip trajectory length was found to be increased with increased level of uncertainty due to stronger lateral deviation from a straight path, accompanied with increased movement duration^28,30^. Based on this evidence, it was emphasized that “the kinematics (trajectory) of the hand can reveal aspects of the cognitive and decision-making process underlying target selection”^29^, reflecting a competition between multiple potential pointing targets during the decision making process and providing evidence for the parallel encoding of the potential actions. Beyond that, the analysis of the time course of movement variability has also provided evidence that movement execution is adapted to uncertainties during target selection^23^ and action planning^31^, i.e. different processes involved in motor decision making, as well as to uncertainty of different origins^23^.

Importantly, each of the processes involved in motor decision making, i.e., the perception of action opportunities, as well as the processes of action selection, planning, and execution, is known to be influenced by developmental changes in later life. In that context, embodied accounts on human behavior emphasize the mutual influence of developmental changes of bodily conditions on an individual's behavior and the interaction with the environment^32,33^. The NFL Framework of Embodied Aging^33^ for example, ascribes age-related slowing in movement initiation partially to both changes in underlying physiological conditions, but also differences in preparatory action planning. Further, developmental perspectives on embodied cognition not only account for decreases in cognitive-motor tasks with increasing age, related to cognitive and sensorimotor aging, but also suggest potential performance benefits with regard to the reactivation of established multimodal associations, which are based on the larger lifetime experience of older adults in interacting with the environment^34^. However, research aiming at further developing and differentiating the theoretical implications of embodied accounts on human behavior in later phases of life, in general, and on the influence of age-related changes in cognitive abilities on the process of motor decision making, including the execution of goal-directed movements, in particular, is still sparse (for exceptions, see^17,18^).

While research into this direction might still be in its early stages, rich empirical evidence on the influence of cognitive aging on cognitive-motor dual-tasking has already been accumulated in the field of human movement science and psychology. Cognitive-motor dual-tasking refers to situations in which individuals have to simultaneously perform a cognitive and a motor task^35^. In these situations, it is commonly observed that performance in either the cognitive and/or motor task is decreased when the resources needed to simultaneously perform both tasks are exhausted due to cognitive aging^35–40^. Consistent with this notion, in a review on the dynamics of sensorimotor-cognitive interdependencies in later life, the increasing investment of cognitive resources for sensorimotor functioning with increasing age has been emphasized^41^. These interdependencies might also become of relevance for everyday life single-task motor behavior as soon as the cognitive resources required to solve that single task exceed available resources e.g., when needing to cognitively process information about dynamically-changing environmental conditions, as during motor decision making under uncertainty. Consequently, the process of motor decision making might be particularly sensitive to cognitive aging, with potential impact on the execution of the motor actions decided upon.

Thus, the aim of this study was to investigate the influence of cognitive aging on motor decision making and execution of an everyday motor task, namely goal-directed pointing. On that account, we presented young and older adults with a motor decision making task, in which participants had to choose between five potential pointing targets. In two conditions, we manipulated the cognitive demands required for understanding the provided information, which indicated the to-be-selected target, to induce varying levels of decision uncertainty. We hypothesized that older adults, due to cognitive aging and general motor slowing^42–46^, would less often select the correct pointing target and execute the goal-directed pointing movements with greater reaction time and longer movement duration as compared to young adults. Further, with respect to existing empirical evidence on motor decision making under uncertainty, we hypothesized that increased cognitive demands during motor decision making would result in increased decision uncertainty in both, young and older adults, as well as changes in spatial movement kinematics, with increased pointing trajectory length^23,28^ as well as increased movement variability across the time course of movement execution^23^. Due to inconclusive results on the influence of decision uncertainty on temporal movement kinematics, i.e. reaction time and movement duration (c.f. Ref.^28^ vs. Ref.^23^), we did not have a directed hypothesis in this regard. Last and most importantly, based on embodied accounts of motor decision making^19^ as well as a developmental embodiment perspective^32,33^, we hypothesized that, due to cognitive aging, increased cognitive demands during motor decision making would result in more pronounced effects on decision uncertainty and movement execution in older adults.

## Methods

The current study was performed as part of a larger project, parts of which have been previously published^47^. As a consequence, the description of “Participants” and “Experimental task and procedure” strongly correspond with that in our previous publication.

### Participants

Thirty-six adults (18 young: mean age ± SD: 23.61 ± 4.41 years, range: 18–32 years, 11 females; 18 older: 66.78 ± 6.09 years, 60–83 years, 10 females) voluntarily participated in the study. Young participants were recruited from a pool of psychology students at University of Tasmania and received either course credit or gift vouchers for their participation. Older participants were recruited through a social media advertisement spread within the greater Hobart area and participated in a draw for one of three gift vouchers as reimbursement. All participants gave written informed consent for participation and potential publication of the study outcomes prior to participation, were right-hand dominant as assessed via the Edinburgh Handedness Inventory^48^, had normal or corrected-to-normal vision, and no known neurological, cognitive or motor impairment that could impact their performance in the study. The experimental procedure was approved by the Human Research Ethics Committee (Tasmania) Network of the University of Tasmania and was in accordance with the principles stated in the Declaration of Helsinki^49^.

### Experimental task and procedure

The study was performed on two days, separated by one week, with a duration of 90–120 min each day. On the first day, all participants performed a cognitive assessment, including the Matrix Reasoning Test, a subtest of the Perceptual Reasoning Index of the Wechsler’s Adult Intelligence Scale IV^50^, as well as COGSTATE computerized cognitive test battery (COGSTATE Ltd., http://www.cogstate.com), which included the following subtests: Groton Maze Chase Test, Groton Maze Learning Test, Detection Task, Identification Task, One Card Learning Task, One Back Task, Two Back Task, Continuous Paired Associate Learning Task, and the Groton Maze Learning Test–Delayed Recall. These tests were performed to determine task-relevant dimensions of cognition, i.e., speed of processing, attention, visuospatial learning and memory, as well as spatial reasoning.

Following the cognitive assessment, whose outcomes have been published previously^47^ and which indicated age-related differences in a subset of tests assessing basic cognitive functions (Groton Maze Chase Test, Detection Task, Identification Task), visuospatial learning and memory (Continuous Paired Associate Learning Task, Two Back Task), as well as executive functions (Groton Maze Learning Test), all participants were familiarized with the experimental task, in which they had to perform goal-directed pointing movements to one of five potential pointing targets of their choice. Specifically, participants were instructed that five circles would appear on the screen, illuminating in a sequential order. Upon an acoustic start signal, they were asked to point towards that circle on the screen, which they assumed would follow next in the sequence. Further, after each trial, they were asked to indicate how confident they are that they pointed towards that circle on the screen, which would indeed follow next in the sequence, on a seven-level Likert-scale, with “1” referring to being very uncertain and “7” referring to being very certain. Decision confidence refers to a particular form of decision (un)certainty, reflecting the subjective belief about the correctness of the decision, given the information uncertainty^51^.

**Figure 1:**
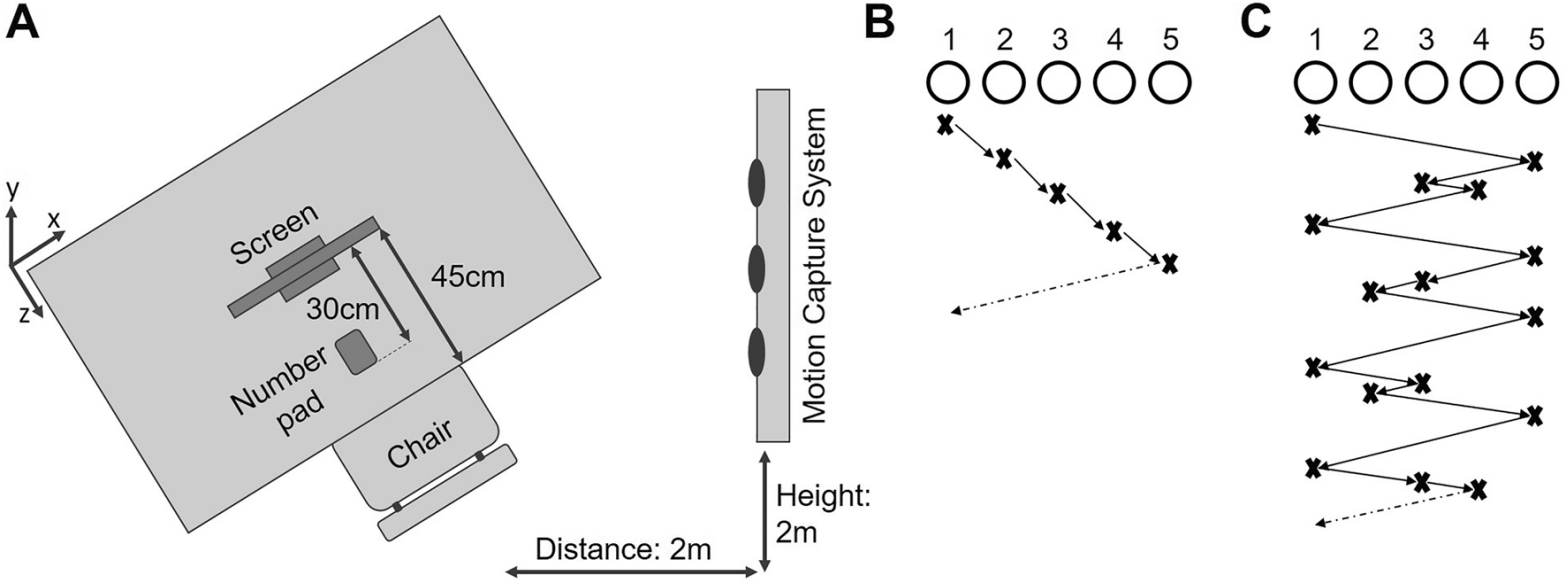
Schematic depiction of the experimental set-up and sequence structures for the two experimental conditions. (**A**) Experimental set-up. Please note that the illustration is not drawn to scale. (**B**) Simple five-element sequence with a sequence pattern following stepwise from left to right. (**C**) Complex 16-element sequence consisting of four different four-element sequence chunks, with each chunk starting with the sequential black filling of the two outermost circles followed by filling of two of the three central circles.

Participants were comfortably seated in front of a 23″ touch screen (Dell P2314Tt), which was placed on a table 45 cm from the edge, with a keyboard positioned 30 cm from the screen (see Fig. 1A), such that they were able to touch the screen without moving the upper body. All participants received written task instructions on the computer screen throughout the experiment, which was controlled through MATLAB R2011a (Mathworks, Natick, MA, United States), using the Psychophysics Toolbox extensions^52,53^. Specifically, at the beginning of each trial, participants were requested to press and hold down a designated start button on a number pad, which was positioned centrally in front of them (see Fig. 1A). Immediately following the button press, five circles (2 cm diameter, 9 cm center-to-center distance between them) were presented on a white background. A sequence was then visually presented to the participants through sequential black filling of the circles. The first element in the sequence was color-coded through the red filling of the circle. Each element was filled for 1 s with no delay between subsequent fillings. After two complete runs of the sequence, the presentation stopped at a pseudo-random position during the third run, with the unfilled circles remaining visible on the screen. After a random delay period of 1–2 s an acoustic signal (450 Hz, 0.2 s duration) informed participants to release the start button and point (i.e., reach and touch) to that circle on the screen that they predicted would be filled next in the sequence. Participants were required to release the start button within 750 ms following the acoustic start signal. If the button release occurred too early (< 100 ms after the acoustic signal) or too late (> 750 ms), the trial was immediately aborted and an error message was presented (“You reacted too early. Please wait for the start tone next time.” or “You reacted too late. Please try to be faster next time.”, respectively), which reminded the participants of the time constraint related to their response initiation. After completion of each pointing movement, participants were asked to indicate their confidence of having pointed to the correct circle. Following the confidence rating, the next trial commenced.

Participants were allowed to practice the task with a much-simplified version, i.e., only three circles on the screen filling sequentially from left to right, until they felt familiar with the procedure and initiated pointing movements consistently within the time constraints. The practice version was composed such that it mirrored the general temporal procedure of both experimental conditions, but was clearly distinct in its visuospatial presentation. None of the participants required more than 20 trials for familiarization. Subsequently, participants performed five blocks of 15 trials each, in which a simple five-element sequence was presented to them. The structure of the sequence was composed in a way that the circles filled orderly from left to right (see Fig. 1B), and was, thus, assumed to be a *simple sequence* with low cognitive demands to decide on the next element in the sequence, resulting in low decision uncertainty i.e., high confidence that the correct pointing target was selected. On the second study day, participants initially had the chance to refamiliarize themselves with the experimental task by use of the practice sequence described above, before performing five blocks of 15 trials each, in which a complex 16-element sequence was presented on the screen (see Fig. 1C). Consequently, the decision about the pointing target, which would follow next in the sequence was based on the knowledge of the *complex sequence* structure, and was thus, supposingly more difficult under the given reaction time constraint as for the simple sequence. Thus, it was assumed that decisions on the pointing target would be performed under higher cognitive demands and increased decision uncertainty i.e., lower confidence that they had selected the correct target. On both days, participants were given the opportunity to rest for a maximum of 5 min between blocks to avoid fatigue-induced changes in task performance. In sum, participants performed goal-directed pointing movements under two experimental conditions: *simple* and *complex*. The order of experimental conditions was not counterbalanced across participants due to organizational reasons: the execution of the simple and complex condition varied in duration due to the different sequence lengths in both conditions. Consequently, performing the complex condition after the initial cognitive assessment would have resulted in a substantially longer first test day. In addition, we assumed the initial cognitive assessment to already be cognitively demanding. Both, testing duration and cognitive demand might have led to mental and/or physical fatigue of the participants, in particular of the older adults. To avoid such potential influence of fatigue on performance in the complex condition, while we assumed the simple condition to be much less sensitive to it, we decided to remain the order of conditions constant across participants.

In every trial, participants could freely choose one out of the five possible pointing targets. Though, in each block of 15 trials, circles 2 and 4 would have been the correct pointing targets in six trials each, with the remaining three trials requiring pointing movements to circles 1, 3 and 5, respectively. To identify the correct pointing target i.e., the circle which would have followed next in the sequence, participants had to be aware of the underlying sequence structure. The level to which participants were aware of the sequence structure may have strongly affected uncertainty during the decision-making process. Thus, to assess the subjectively perceived uncertainty, participants were asked to indicate their confidence on whether they believed they pointed towards the correct target on a seven-level Likert-scale after every trial.

To investigate the influence of cognitive aging and decision uncertainty on the execution of goal-directed pointing movements, fingertip trajectories were recorded at a recording frequency of 100 Hz using a three-camera optical motion tracking system (Optotrak 3020, Northern Digital Inc., US). On that account, two markers were attached on the right index finger, with one marker placed centered on the fingernail and the second one on the skin directly below the fingernail.

### Data processing and analysis

Data processing and analysis was performed using customized MATLAB scripts with kinematic data analysis following the processing and analysis procedure of previous work of the group^23^: Three-dimensional fingertip trajectory data was processed to analyze spatial movement characteristics. Maximum endpoint location of the fingertip in the depth-direction was identified for both markers, de facto being bounded by the location of the touch screen (see Fig. 1A). The corresponding sampling point was defined as movement offset. Subsequently, movement velocity in the depth direction was calculated as the first derivative of the fingertip trajectory with respect to time and maximum velocity was identified. Movement onset was then defined as the last sampling point before exceeding 5% of maximum velocity. After the definition of movement onset and offset, it was checked, which of the two fingertip markers provided the most valid sampling points i.e., the smallest number of missing values. This fingertip marker was then selected for further processing. Following this selection, trials in which fingertip position at movement onset and offset deviated more than 2 cm from median fingertip position at the respective time point, were identified as outliers and excluded from further analyses. Only trials with pointing movements to either circles 2 or circle 4 were analyzed, as those represented the most trials (see “Experimental task and procedure” above) and were of equal distance to the virtual line between the start button and the center of the screen, thus, being eligible for being collapsed across during kinematic data analysis. Following parameters were calculated on the remaining valid trials (all participants: mean n_trials_ ± SD: 32.86 ± 12.45), separately for each participant:

First, as a measure of *task performance,* the percentage of correct pointing movements i.e., the number of correctly indicated circles, was calculated across all valid trials. Second, as an indirect measure of decision uncertainty, representing the subjectively perceived uncertainty^4,5^, *decision confidence* was calculated as the mean confidence level, averaged across all valid trials. To account for the known close relationship between error processing and confidence judgement in decision making^12–14^, as well as empirical evidence on age-related changes in error-awareness^9^, decision confidence and all further parameters were analyzed once for all valid trials, i.e. collapsed across trials of correct and incorrect pointing movements, as well as separately for correct trials, only. Please note that only correct but not incorrect pointing movements were analyzed separately, as particularly in the simple condition, the expected low number of incorrect trials would not permit any statistical analysis.

Two parameters quantifying temporal movement characteristics were calculated to assess the execution of the goal-directed pointing movements. First, mean *reaction time* was calculated as the time difference between the acoustic start signal and start button release, averaged across all valid trials. Second, mean *movement duration* was calculated as the time difference between start button release and first contact with the touch screen, averaged across all valid trials.

Finally, three parameters quantifying spatial movement characteristics were calculated: *path length*, *time course of variability in fingertip position*, as well as *endpoint variability in fingertip position*. First, the distance traveled between two successive sampling points was calculated as the square-root of the summed squared distances traveled in horizontal and vertical direction. Depth direction was not considered in this calculation as it was predefined by the experimental set-up and kept constant throughout the experiment. *Path length* was then calculated as the sum of the traveled distance across all successive sampling points in each trial, normalized to the shortest distance between fingertip position at movement onset and offset. Next, to be able to analyze the *time course of variability in fingertip position*, fingertip trajectories were space-normalized to 11 equidistant sampling points between movement onset and offset, referring to 0–100% in 10%-steps of traveled distance in depth direction. Space-normalization of movement trajectories was preferred over time-normalization, as we hypothesized differences in temporal movement characteristics between young and older adults, which impairs the adequacy of time-normalization (for a more detailed explanation, see^28^). Subsequently, the *time course of variability in fingertip position* was calculated as the within-subject between-trial standard deviation of the mean horizontal and vertical fingertip position at each of the 11 pointing samples, separately for both target positions in each condition, following the procedure of previous work of our group^23,54^. Standard deviations were subsequently averaged across both target positions. Finally, variability at the last pointing sample of the above-described time course was defined as *endpoint variability in fingertip position*.

### Statistical analysis

All statistical analyses were performed using JASP, Version 0.16.3^55^ and descriptives will be provided as mean ± standard deviation. Following initial exploration of the data, Block 1 was not considered for further statistical analyses, as task performance in the complex *C*ondition was strongly affected by learning effects i.e., the identification and learning of the underlying sequence structure. Remaining trials were merged across blocks. Participants whose scores were ± 1.5 times the interquartile range were considered as outliers and excluded from further analyses.

Given that the simple condition was composed to impose a low cognitive demand, we assumed task performance and decision confidence in this condition to show a ceiling effect and low variance in both age groups. Thus, to be able to statistically analyze potential age-related differences between both conditions, the within-subject *difference in task performance* as well as the within-subject *difference in decision confidence* between simple and complex conditions was calculated. Subsequently, t-test for independent samples (*Age group*: young vs. older adults) was conducted on the difference in task performance and difference in decision confidence. Mann–Whitney-U-test was conducted, in case of non-normal distribution of the data.

A 2 × 2 mixed-factor ANOVA was conducted for decision confidence, reaction time, movement duration, path length and endpoint variability of fingertip position with *Age group* (young vs. older adults) as a between-subject factor and *Condition* (simple vs. complex) as a repeated factor. Significant interactions between Age group and Condition were followed up by separate t-tests for post-hoc analyses. Further, a 2 × 2 × 11 mixed-factor repeated measures ANOVA with Age group as between-subject factor, and Condition as well as Pointing sample as repeated factors was run on variability of fingertip position. Significant interactions between Condition and Pointing sample were followed up by paired-sample t-tests for post-hoc analyses. The critical level of statistical significance was set to 0.05. Greenhouse–Geisser corrections were applied if the assumption of sphericity for an ANOVA was violated. Effect sizes (*η*_*p*_^*2*^ and *Cohen’s d*) were calculated to aid in the interpretation of the magnitude of observed effects. In accordance with Cohen^56^, *η*_*p*_^*2*^ ≥ 0.06 was considered as medium effects and *η*_*p*_^*2*^ ≥ 0.14 as large effects. Further, 0.2 <|*Cohen’s d*|≤ 0.5 was considered a small effect, 0.5 <|*Cohen’s d*|≤ 0.8 as medium effect and |*Cohen’s d*|> 0.8 was considered a large effect.

## Results

### Task performance

**Figure 2:**
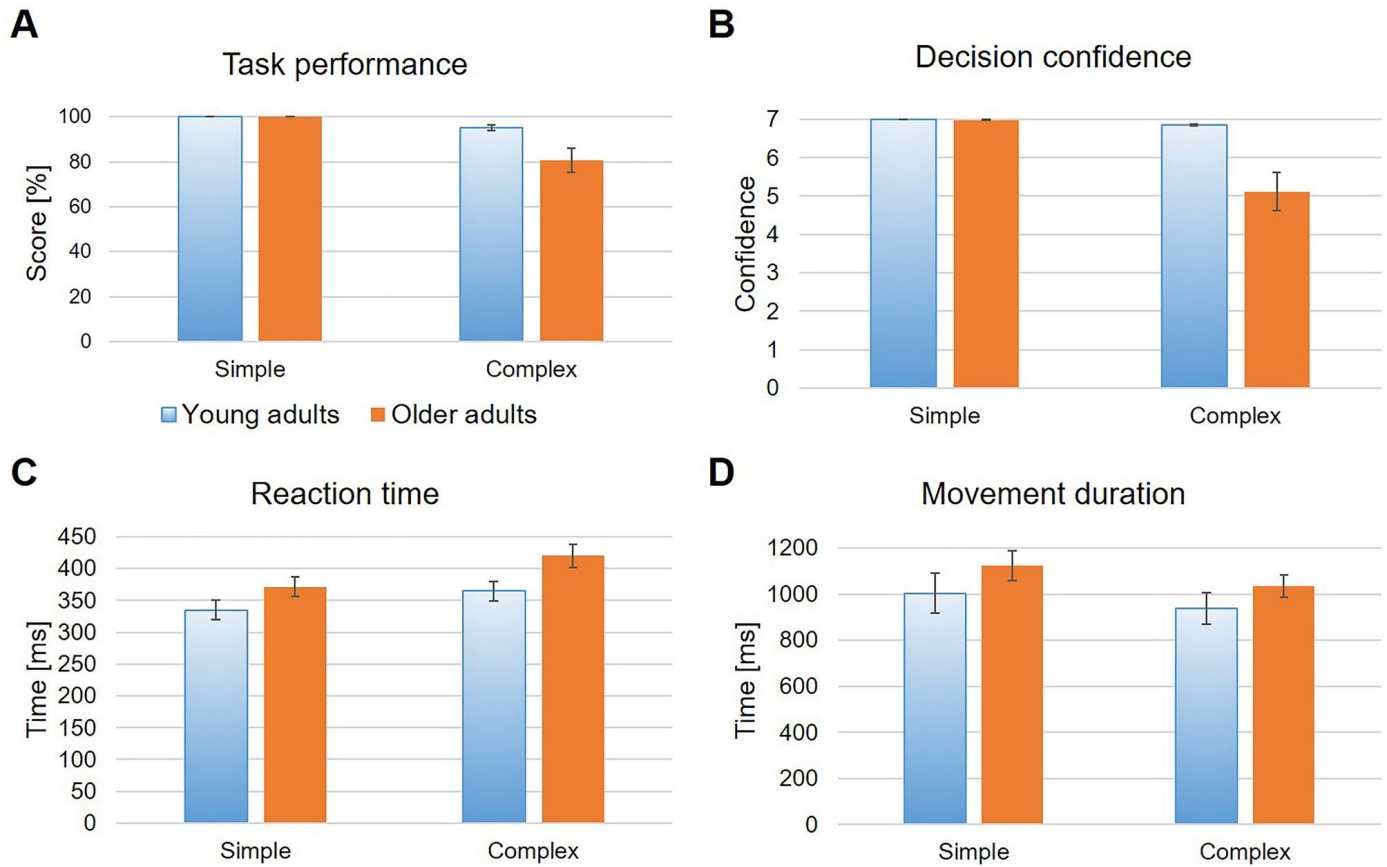
Behavioral outcomes for young and older adults in both experimental conditions. Blue, shaded bars represent young adults’ outcomes while orange, filled bars represent older adults’ behavioral outcomes. For all parameters mean values ± SEM for all valid trials are depicted. (**A**) Task performance, calculated as the percentage of targets correctly pointed at. (**B**) Decision confidence i.e., perceived uncertainty about whether participants pointed at the correct target, as indicated on a seven-level Likert-scale after each pointing movement. (**C**) Reaction time between start signal and start button release. (**D**) Movement duration between start button release and first contact with the touch screen.

Both young and older adults showed best possible task performance, i.e. always pointed towards the correct target, in the simple sequence condition (Fig. 2A). Task performance was lower in both young and older adults when indicating the next sequence element after complex sequence presentation, with 95.01 ± 4.92% and 80.65 ± 22.63% correctly indicated targets, respectively. Consequently, older adults showed a stronger decrease in task performance than young adults. Precisely, the difference in task performance between simple and complex conditions was 4.18 ± 4.00% and 18.26 ± 22.88% for young and older adults, respectively, and was significantly different between Age groups with large effect size, *t*(28) = 62.50, *p* = 0.04, *Cohen’s d* = − 0.83.

### Decision confidence

Confidence about the correctness of the pointing decision was maximal for both age groups in the simple condition, with mean confidence level of 7.00 ± 0.00 for young and 6.98 ± 0.03 for older adults, respectively (see Fig. 2B). Both young and older adults indicated lower decision confidence in the complex condition, with a mean confidence level of 6.85 ± 0.11 for young and 5.11 ± 2.12 for older adults, and a mean difference in decision confidence of 0.01 ± 0.11 and 1.53 ± 2.01 for young and older adults, respectively. This difference in the change of decision confidence between Age groups was statistically significant with medium effect size, *t*(25) = 34.00, *p* = 0.007, *Cohen’s d* = − 0.62. When considering correct trials, only, this main effect of Age group disappeared, *t*(29) = 73.00, *p* = 0.07, *Cohen’s d* = − 0.39, due to small decreases in decision confidence for young adults and small increases in decision confidence for older adults in the complex condition. Specifically, mean confidence levels were 6.60 ± 0.63 for young and 5.23 ± 2.10 for older adults, while decision confidence remained unchanged for both age groups in the simple condition. Consequently, mean differences in decision confidence also changed and were 0.43 ± 0.67 and 1.40 ± 2.00 for young and older adults, respectively.

### Movement kinematics

#### Temporal movement characteristics

Generally, young adults exhibited faster reaction times than older adults, with mean reaction times of 334.80 ± 65.97 ms and 364.37 ± 64.24 ms for young adults, and 371.24 ± 64.75 ms and 419.90 ± 79.35 ms for the older adults in the simple and complex condition, respectively (see Fig. 2C). This age-related difference in mean reaction times was statistically significant with a medium effect size, *F*(1,34) = 4.53, *p* = 0.04, *η*_*p*_^*2*^ = 0.12. Further, the main effect of Condition was significant, *F*(1,34) = 25.87, *p* < 0.001, *η*_*p*_^*2*^ = 0.43, and was associated with a large effect size. The interaction of Age group × Condition was not statistically significant, *F*(1,34) = 1.55, *p* = 0.22, *η*_*p*_^*2*^ = 0.04. Analyses conducted using correct trials, only, yielded the same statistical results (see Supplementary Table 1), due to only minor changes in mean reaction times with 335.01 ± 66.37 ms and 359.17 ± 61.36 ms for young adults, and 371.26 ± 64.77 ms and 411.37 ± 73.50 ms for older adults in the simple and complex condition, respectively.

Movement duration was qualitatively similar for both age groups and experimental conditions (see Fig. 2D). Young adults executed their goal-directed pointing movements with an average movement duration of 1003.57 ± 366.64 ms for the simple and 937.86 ± 280.46 ms for the complex condition. Older adults executed their pointing movements with an average movement duration of 1125.04 ± 271.65 ms for the simple and 1034.18 ± 200.46 ms for the complex condition. Movement duration was not significantly different between either Age groups, *F*(1,32) = 1.85, *p* = 0.18, *η*_*p*_^*2*^ = 0.06, Conditions, *F*(1,32) = 1.21, *p* = 0.28, *η*_*p*_^*2*^ = 0.04, or in the interaction of Age group × Condition, *F*(1,32) = 0.25, *p* = 0.62, *η*_*p*_^*2*^ < 0.01. Again, the separate analysis of correct trials, only, resulted in the same outcomes (see Supplementary Table 1), due to only minor changes in mean movement durations with 1013.13 ± 379.72 ms and 933.20 ± 282.24 ms for young adults, and 1127.69 ± 269.60 ms and 1026.72 ± 200.51 ms for older adults in the simple and complex condition, respectively.

#### Spatial movement characteristics

**Figure 3:**
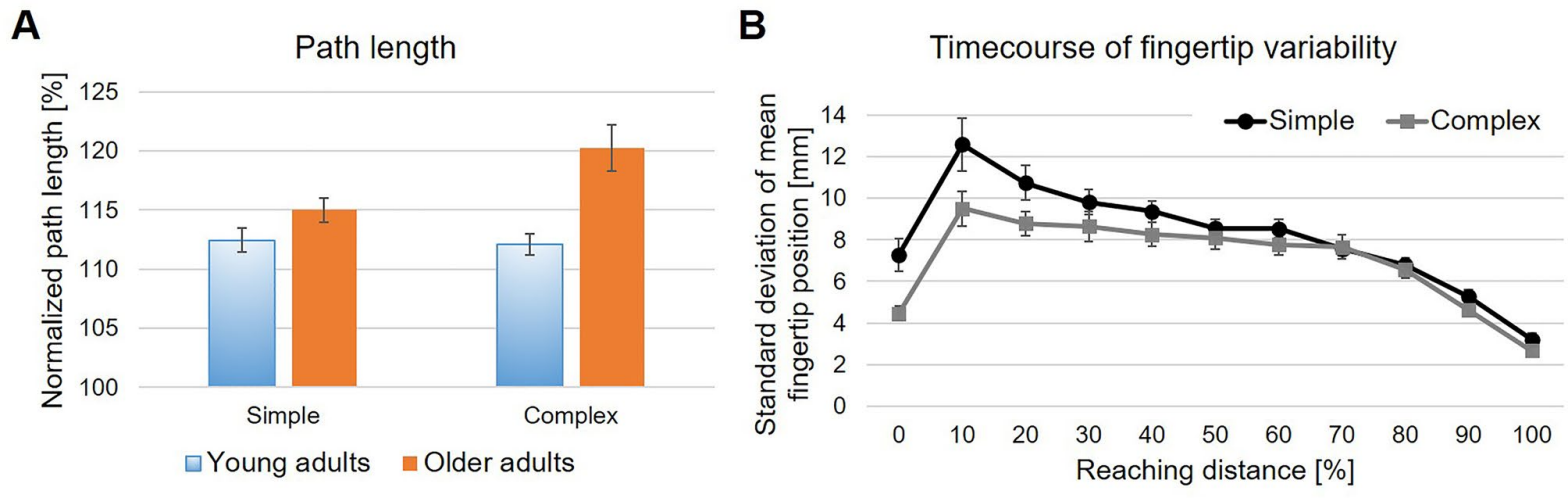
Spatial movement characteristics. For both parameters mean values ± SEM are depicted. (**A**) Path length normalized to straight path length between fingertip position at movement onset and offset. (**B**) Time course of variability in fingertip position for both experimental conditions.

Both young and older adults executed pointing movements which deviated from the straight line between fingertip positions at movement onset and offset in both experimental conditions. While mean path length was 112.44 ± 4.03% and 112.08 ± 3.80% relative to straight path length for the young adults in the simple and complex condition, respectively, older adults’ mean path length was 114.99 ± 2.89% and 120.23 ± 8.14%, respectively (see Fig. 3A). The qualitative observation of increased path length in older adults was supported by a statistically significant main effect of Age group with large effect size, *F*(1,20) = 7.03, *p* = 0.02, *η*_*p*_^*2*^ = 0.26. Besides, path length was not statistically different between Conditions, *F*(1,20) = 0.28, *p* = 0.60, *η*_*p*_^*2*^ = 0.01, nor in the interaction of Age group × Condition, *F*(1,20) = 1.35, *p* = 0.0.26, *η*_*p*_^*2*^ = 0.06. When analyzing correct trials, only, mean path length only minimally changed, with 112.46 ± 4.01% and 112.99 ± 5.01% for young adults, and 114.99 ± 2.89% and 120.16 ± 8.23% for older adults in the simple and complex condition, respectively. No significant differences became evident, neither between Age group, Condition nor in the interaction of Age group × Condition (see Supplementary  Table 1 for complete statistical values).

The time course of variability in fingertip position showed a steep increase in variability at the very beginning of the movement and a subsequent continuous decrease in variability, with no differences between conditions at movement end (see Fig. 3B). This qualitative description is supported by a significant main effect of Pointing sample with large effect size, *F*(2.29,38.99) = 20.24, *p* < 0.001, *η*_*p*_^*2*^ = 0.54. Bonferroni-corrected post-hoc comparisons of single Pointing samples are reported in Table 1. In addition, variability in fingertip position was significantly less in the complex condition as compared to the simple condition, as indicated by the significant main effect of Condition with large effect size, *F*(1,17) = 5.19, *p* = 0.04, *η*_*p*_^*2*^ = 0.23. The interaction of Condition × Pointing sample was also significant with large effect size, *F*(2.80,47.51) = 2.96, *p* = 0.045, *η*_*p*_^*2*^ = 0.15. Post-hoc pairwise comparisons of differences between conditions at single pointing samples revealed that variability of fingertip position was significantly less in the complex condition in the first half of the pointing movement, i.e. until around 40% of the reaching distance, all with medium effect size, with no differences afterwards. Detailed statistical values are reported in Table 2. Importantly, young and older adults did not differ significantly in the variability of fingertip position, as neither the main effect of Age group, nor any interaction effect involving Age group was significant, including the three-way interaction of Age group × Condition × Pointing sample, all *p* ≥ 0.27 (see Supplementary  Table 2 for complete statistical values). When analyzing correct trials, only, the outcomes of the statistical analyses were slightly different: First, the main effect of Condition was not significant, *F*(1,17) = 4.19, *p* = 0.056, *η*_*p*_^*2*^ = 0.20, while the interaction effect of Condition × Pointing sample remained significant with large effect size, *F*(3.50,59.49) = 4.92, *p* = 0.003, *η*_*p*_^*2*^ = 0.17. The main effect of Pointing sample was also significant with large effect size, *F*(2.56,43.44) = 24.42, *p* < 0.001, *η*_*p*_^*2*^ = 0.59. Further, while the main effect of Age group was still not significant, both age groups showed significantly different variability in fingertip position across the time course of movement execution, as indicated by the significant interaction effects of Age group × Pointing sample, *F*(2.56,43.44) = 3.59, *p* = 0.03, *η*_*p*_^*2*^ = 0.17, as well as of Age group × Condition × Pointing sample, *F*(3.50,59.49) = 3.37, *p* = 0.02, *η*_*p*_^*2*^ = 0.01, with large and small effect size, respectively (see Supplementary Table 3 for complete statistical values). Post-hoc analysis revealed that these interaction effects could be assigned to significantly lower variability of fingertip position for young adults in the complex condition as compared to older adults in the simple condition at the very beginning of the pointing movement, and lower variability of fingertip position of older adults in the complex condition as compared to young adults in both conditions at 10% of the reaching distance (see Supplementary Table 4 for complete statistical values as well as Supplementary Fig. 1 for a graphical depiction). No further differences in variability of fingertip position for either combination of Age group and Condition at individual Pointing samples turned out to be significant. Last, the analysis of endpoint variability of fingertip position did not reveal any significant effect, all *p* ≥ 0.31 (see Supplementary  Table 5 for complete statistical values).

**Table 1:** Post-hoc comparisons of variability in fingertip position at single pointing samples. Bonferroni-corrected p-values of significant effects are reported.

		Pointing sample [%]									
		10%	20%	30%	40%	50%	60%	70%	80%	90%	100%
Pointing sample [%]	0%	*p* < 0.001	*p* < 0.001	*p* = 0.003	*p* = 0.003	*p* = 0.009	n.s.	n.s.	n.s.	n.s.	*p* < 0.001
	10%		n.s.	n.s.	n.s.	n.s.	n.s.	n.s.	*p* < 0.001	*p* < 0.001	*p* < 0.001
	20%			n.s.	n.s.	n.s.	n.s.	n.s.	*p* = 0.009	*p* < 0.001	*p* < 0.001
	30%				n.s.	n.s.	n.s.	n.s.	*p* = 0.03	*p* < 0.001	*p* < 0.001
	40%					n.s.	n.s.	n.s.	*p* = 0.04	*p* < 0.001	*p* < 0.001
	50%						n.s.	n.s.	n.s.	*p* < 0.001	*p* < 0.001
	60%							n.s.	n.s.	*p* < 0.001	*p* < 0.001
	70%								n.s.	*p* = 0.002	p < 0.001
	80%									n.s.	*p* < 0.001
	90%										n.s.

**Table 2:** Post-hoc comparisons of variability in fingertip position between conditions at single pointing samples.

Pointing sample	*t*	*df*	*p*	*Cohen’s d*
0%	4.01	29	< 0.001***	0.73
10%	2.55	30	0.02*	0.46
20%	2.79	27	0.01*	0.53
30%	3.13	25	0.004**	0.62
40%	2.41	26	0.02*	0.46
50%	1.01	27	0.32	0.19
60%	1.20	29	0.24	0.22
70%	0.04	28	0.97	0.01
80%	0.86	28	0.40	0.16
90%	1.86	28	0.07	0.35
100%	1.09	29	0.29	0.20

Significant at a level of *p < 0.05, **p < 0.01, ***p < 0.001.

## Discussion

In this study, we investigated the influence of cognitive aging on uncertainty during motor decision making and execution of an everyday motor task, namely goal-directed pointing, in two experimental conditions of varying cognitive demands. To manipulate decision uncertainty in young and older adults, we presented visuospatial sequences of two different complexities, composed by five sequentially filling circles on a computer screen. Following sequence presentation, participants had to perform a pointing movement towards that circle on the screen which they assumed to follow next in the sequence. Consequently, selection of the correct pointing target was dependent on an understanding of the sequence structure. Previously, we have been able to show that performance in this task relies on cognitive functioning related to the processing and storage of sequence information, which was found to be sensitive to cognitive aging, as well as problem solving abilities^47^. We hypothesized that increased cognitive demands during motor decision making, manipulated through the complexity of the visuospatial sequence structure, would lead to greater decision uncertainty, due to inadequate understanding of available information for pointing target selection, with consequences for subsequent movement execution. More importantly, we also hypothesized that increased cognitive demands during motor decision making would result in more pronounced effects on decision uncertainty and movement execution in older as compared to young adults, due to cognitive aging.

First of all, we found both aging and cognitive demands to independently affect execution of goal-directed pointing movements. Thereby, the finding of increased reaction time in older as compared to young adults, as observed in our study, provides further empirical evidence for a general motor slowing with increasing age, as also commonly suggested in the literature^45,46^. However, movement duration was not increased in older adults. Further, in the current study, we found significantly increased path length in older as compared to young adults, which might be explainable as longer fingertip trajectories reflecting the ongoing competition between to-be-selected pointing targets^28,57^. Interestingly, this effect disappeared when only correct pointing movements were considered. This suggests that the competition of potential actions was solved before movement execution in trials in which the correct pointing target was selected, while the ongoing competition had pronounced effects on path length in the lower number of incorrect trials, such that it affected statistical parameters when collapsed across all valid trials. However, to directly prove this assumption an experimental set-up has to be developed that allows to record a sufficiently high number of both correct and error trials to be able to also run separate analyses on the latter.

On the other hand, cognitive demands during motor decision making, as manipulated through sequence complexity, affected temporal and spatial movement parameters independently of age. Specifically, both young and older adults required longer reaction times to initiate the movement after complex sequence presentation. This finding is in line with previous research by our group^23^, but in contrast to further empirical evidence which found reaction time to be unaffected by manipulations of uncertainty during motor decision making^28^. Various reasons might account for this difference. First, the reaction time constraint applied in our study (750 ms) was less strict than in other experimental set-ups (325 ms)^28^. The less-strict reaction time constraint in the current study was chosen to allow older adults to also meet the reaction time constraint, given the above-mentioned well-established general motor slowing with increasing age^42,45,46^. Naturally, a less-strict reaction time constraint provided more freedom to exploit the available time e.g., when needing more time to process visual-spatial sequence information to decide upon the pointing target. Second, the manipulation of decision uncertainty, itself, might, at least partially, explain the divergence between existing empirical evidence and our study results. This explanation will be further elaborated on in the last paragraph of this section, below.

Interestingly, we found increased cognitive demands during motor decision making to also affect spatial movement parameters (see Fig. 3), though, in a direction opposite to what we had hypothesized. Based on existing empirical evidence, we hypothesized to find increased path length^23,28–30^ as well as increased variability in fingertip position^23^ in the complex condition. In contrast to that, we found lower fingertip variability across the time course of movement execution in the condition of higher decision uncertainty i.e., in the complex condition, and no differences in path length between experimental conditions. This finding seems to be counterintuitive at first, but might be explained when also considering the observed increased reaction time in the complex condition. We hypothesized a-priori that increased path length and variability of fingertip position would be indicative of an ongoing competition between multiple potential pointing targets^28,30^. Instead, participants might have exploited the available time before movement onset to accumulate additional evidence for the then selected target^25,58,59^. Thus, when executing the pointing movement, movement execution occurred under lower competition between alternative action opportunities. However, while this line of reasoning might provide an explanation why we did not find empirical evidence for our hypothesis, it remains to be investigated why variability in of fingertip position is even decreased under higher cognitive demand. Further research trading off decision time i.e., reaction time, and levels of decision uncertainty would be required to follow up on this question.

The main objective of this study was to investigate the interaction of age and cognitive demands on motor decision making and the execution of goal-directed pointing movements. The analysis revealed significant differences in the change of task performance and decision confidence from simple to complex condition between young and older adults. While both age groups showed optimal performance, i.e. always pointed towards the correct target, and indicated maximal confidence about the correctness of their pointing decision under low cognitive demand, older adults showed pronounced decrements in both parameters under higher cognitive demand during motor decision making. It is important to note that the difference in confidence rating between conditions of low and higher cognitive demand was not significantly different between age groups when considering correct trials, only. This finding is in line with the notion of a close relation of error monitoring and confidence judgement^12–14^, suggesting higher confidence ratings for correct trials and lower confidence ratings for incorrect trials. Consequently, different confidence ratings were to be expected between both age groups when taken into account that older adults pointed less often towards the correct target, i.e. produced more errors, in the complex condition, but not when excluding incorrect trials form the analysis. However, this finding is not in line with recent empirical evidence suggesting decreased error awareness with increasing age^9^, though further research, including neurophysiological recording techniques, is necessary to investigate to which extent older adults were aware of incorrected decisions.

Importantly, no interaction effects of age and cognitive demand were found for any of the kinematic parameters, besides a very specific interaction effect of age and cognitive demand on variability in fingertip position at the beginning of the pointing movement, i.e. until 10% of the reaching distance. Since this interaction became evident in only three out of the 66 relevant comparisons between young and older adults in the simple and complex condition and at individual sampling points over the time course of movement execution, this effect should be not be overinterpreted but treated with caution.

Taken together, the findings provide a differentiated picture of age-related changes in motor decision making under uncertainty, due to inadequate understanding of available information, and related movement execution: Recent theoretical works emphasize an embodied account on perceptual and motor decision making^19–21,60^, suggesting a mutual influence of decision and motor processes in motor decision making. This assumption is increasingly supported by empirical evidence, showing that the characteristics of the motor action influence the decision process^61–63^, and suggesting a coregulation of decision and motor processes, potentially due to shared neural mechanisms^25,26^. Importantly, developmental embodiment perspectives suggest age-related changes in the links between perception, cognition and action^32–34^. In the current study we indeed found interaction effects of age and cognitive demand on the decision process, i.e. task performance and decision confidence, but different, independent effects of age and cognitive demand on spatial and temporal aspects of movement execution, i.e. reaction time, path length and variability in fingertip position. Thus, our findings are partially in accordance with embodiment accounts on human decision making, though further research is needed to be able to carve out the causes of the divergent findings in our study and their theoretical implications.

On the one hand, independent effects of age and cognitive demand on movement execution following decision making under uncertainty might be indicative for independent mechanisms mediating the effects of age and cognitive demand on the motor processes during motor decision making. On the other hand, in particular embodiment accounts on action emphasize the complex interconnectedness of neural, cognitive, motor functional and behavioral changes across the lifespan^32–34^. Recent research on (motor) decision making in older adults^6,15–18^ provided evidence that older adults adjust their decision strategies to compensate for the age-related changes in the available (cognitive) resources required to cope with decision uncertainty. Thus, while in young adults, motor decision making under uncertainty seems to be characterized by a coregulation of decision and motor processes^25,28^, aging might increase the complexity of coping mechanisms by involving additional strategic behavioral changes, not detected in the current study. To disentangle between both alternatives, experimental set-ups need to be developed, which allow the manipulation of motor demands^25,26^, and which also allow to analyze motor processes at different levels of the human motor system e.g. neural and muscle activity, synergistic movement coordination^64,65^, under varying cognitive demands in young and older adults.

Finally, in the current study, to be able to investigate the effect of cognitive aging on motor decision making and subsequent movement execution, we targeted a different origin of decision uncertainty as in previous research. While common experimental set-ups usually provoke decision making under risk, in which not all information necessary for action selection are available at movement onset^18,28,30^ (i.e. incomplete information), or provide ambiguous choice options^23^ (i.e. undifferentiated alternatives), in the current study we provided all necessary information before movement onset, but manipulated the cognitive demands required to interpret this information (i.e. inadequate understanding). We hypothesized that this manipulation would induce different levels of decision uncertainty and affect execution of the decision outcome. This different origin of decision uncertainty might have differently impacted temporal and spatial movement kinematics. In line with this assumption, we found decreased fingertip variability across the time course of movement execution under increased cognitive demands during motor decision making, while previous research of our group found increased fingertip variability with increasing level of decision uncertainty^23^. In sum, this suggests that, while decision uncertainty induced by *incomplete information, undifferentiated alternatives* or *inadequate understanding* induce behavioral effects which reflect in the execution of the decision outcome, these effects differ in their quality. Thus, further research is needed to investigate how different origins of uncertainty differently affect the interplay of cognitive and sensorimotor processes during action selection, planning, and execution.

Last, in this study, we investigated the influence of cognitive demand on motor decision making and movement execution in an everyday, yet still complex motor task, namely goal-directed pointing. While recent theoretical work in psychology plead for a stronger consideration of action in the study of psychological processes^20,60,66^, its implementation into research practice comes with meaningful consequences for the experimental set-up and procedure, e.g. with regard to testing order of different conditions, learning effects, as well as number of trials. In the current study, using a complex motor task, participants performed 75 trials per condition, of which maximally 48 were analyzed (see “Participants” and “Experimental task and procedure”). This number is substantially lower as in typical perceptual decision making studies using saccades or button presses as responses^61^, with the number of trials ranging in the hundreds. Still, each experimental session lasted about 60–90 min, thus, experimental duration is a constraining factor for the number of trials to be recorded using complex motor tasks. As a consequence of this, analyses typically performed in decision making research, e.g. the separate analysis of correct and error trials^67,68^, was not possible and would not be easy to implement in the future. Developing experimental set-ups which allow the production of complex movements as the outcome of decision making under variable cognitive and motor demands, remains a challenge for the future, with recent research having taken up the challenge^22,26^.

## Conclusion

In summary, the current study indicates that both age and cognitive demands for interpreting available information have independent as well as interacting effects on motor decision making. While the independent effects of age and cognitive demands manifest themselves in age- as well as cognitive demand-related differences in movement execution, the interaction of both becomes evident in the decision process, quantified as task performance and decision uncertainty. The outcomes of this study open up interesting new starting points for future research regarding the coregulation of decision and motor processes during motor decision making in young and older adults.

### Supplementary Information

Supplementary Information.

## Data Availability

Anonymized data is available on OSF (10.17605/OSF.IO/QSFG4).

## Code availability

Customized analytical scripts are available on OSF (10.17605/OSF.IO/QSFG4).
