# Supplementary material for: Influence of age and cognitive demand on motor decision making under uncertainty: a study on goal directed reaching movements
Source: Sci Rep. 2024 Apr 20;14:9119. doi: 10.1038/s41598-024-59415-7 (PMC11032380; doi:10.1038/s41598-024-59415-7)
Supplement: Supplementary file 1 — Supplementary Information. [file 41598_2024_59415_MOESM1_ESM.docx]

Supplementary Material

**Supplementary table 1** – Statistical values for main and interaction effect analyses of temporal and spatial movement parameters, including correct trials, only.

|  | ***df*** | ***F*** | ***p*** | ***η_p_²*** |
| --- | --- | --- | --- | --- |
| **Reaction time** | | | | |
| ***Age group*** | *1* | *4.46* | *0.04* | *0.12* |
| *Residuals* | *34* |  |  |  |
| ***Condition*** | *1* | *18.74* | *< 0.001* | *0.36* |
| *Residuals* | *34* |  |  |  |
| ***Age group × Condition*** | *1* | *1.16* | *0.29* | *0.03* |
| *Residuals* | *34* |  |  |  |
| **Movement duration** | | | | |
| ***Age group*** | *1* | *1.66* | *0.21* | *0.05* |
| *Residuals* | *32* |  |  |  |
| ***Condition*** | *1* | *1.97* | *0.17* | *0.06* |
| *Residuals* | *32* |  |  |  |
| ***Age group × Condition*** | *1* | *0.19* | *0.67* | *0.01* |
| *Residuals* | *32* |  |  |  |
| **Path length** | | | | |
| ***Age group*** | *1* | *2.56* | *0.12* | *0.11* |
| *Residuals* | *21* |  |  |  |
| ***Condition*** | *1* | *0.54* | *0.47* | *0.03* |
| *Residuals* | *21* |  |  |  |
| ***Age group × Condition*** | *1* | *0.55* | *0.47* | *0.03* |
| *Residuals* | *21* |  |  |  |

**Supplementary table 2** – Statistical values for main and interaction effects involving Age group for variability in fingertip position referring to all valid trials.

|  | ***df*** | ***F*** | ***p*** | ***η_p_²*** |
| --- | --- | --- | --- | --- |
| ***Age group*** | *1* | *0.645* | *0.43* | *0.04* |
| *Residuals* | *17* |  |  |  |
| ***Age group × Condition*** | *1* | *0.01* | *0.91* | *< 0.001* |
| *Residuals* | *17* |  |  |  |
| ***Age group × Sample*** | *2.29ᵃ* | *0.63ᵃ* | *0.56ᵃ* | *0.01* |
| *Residuals* | *38.99ᵃ* |  |  |  |
| ***Age group × Condition × Sample*** | *2.80ᵃ* | *1.36ᵃ* | *0.27ᵃ* | *0.006* |
| *Residuals* | *47.51ᵃ* |  |  |  |

^a^ Greenhouse-Geisser corrected values

**Supplementary table 3** – Statistical values for main and interaction effect analyses of variability in fingertip position referring to correct trials, only.

|  | ***df*** | ***F*** | ***p*** | ***η_p_²*** |
| --- | --- | --- | --- | --- |
| **Variability in fingertip position** | | | | |
| ***Age group*** | *1* | *0.02* | *0.89* | *0.001* |
| *Residuals* | *17* |  |  |  |
| ***Condition*** | *1* | *4.19* | *0.06* | *0.20* |
| *Residuals* | *17* |  |  |  |
| ***Sample*** | *2.56^a^* | *24.42 ^a^* | *< 0.001 ^a^* | *0.59* |
| *Residuals* | *43.44 ^a^* |  |  |  |
| ***Age group × Condition*** | *1* | *0.32* | *0.58* | *0.02* |
| *Residuals* | *17* |  |  |  |
| ***Age group × Sample*** | *2.56^a^* | *3.59 ^a^* | *0.03 ^a^* | *0.17* |
| *Residuals* | *43.44 ^a^* |  |  |  |
| ***Condition × Sample*** | *3.50 ^a^* | *4.92 ^a^* | *0.003 ^a^* | *0.02* |
| *Residuals* | *59.49 ^a^* |  |  |  |
| ***Age group × Condition × Sample*** | *3.50 ^a^* | *3.37 ^a^* | *0.02 ^a^* | *0.01* |
| *Residuals* | *59.49 ^a^* |  |  |  |

^a^ Greenhouse-Geisser corrected values

**Supplementary table 4** – Bonferroni corrected post-hoc comparisons of variability in fingertip position for the three-way interaction Age Group × Condition × Pointing sample for correct trials only. Only statistical values for significant differences of comparisons within individual sampling points are presented. Significant effects spanning different pointing samples are neglected. All other comparisons are non-significant.

| ***Pointing sample*** | ***Age group × Condition*** | ***Mean difference*** | ***SE*** | ***t*** | ***p*** |
| --- | --- | --- | --- | --- | --- |
| ***0%*** | *older adults_simple_ vs.*  *young adults_complex_* | *4.77* | *1.124* | *4.25* | *0.04** |
| ***10%*** | *young adults_simple_ vs.*  *older adults_complex_* | *4.77* | *1.124* | *4.25* | *0.04** |
| ***10%*** | *young adults_complex_ vs.*  *older adults_complex_* | *5.48* | *1.124* | *4.88* | *0.003*** |

*significant at a level of * p < .05, ** p < .01*

**Supplementary table 5** – Statistical values for main and interaction effect analyses of endpoint variability in fingertip position, separately for the two analyses including all valid trials, i.e. both correct and incorrect trials, as well as correct trials, only.

|  | ***df*** | ***F*** | ***p*** | ***η_p_²*** |
| --- | --- | --- | --- | --- |
|  | **All valid trials** |  |  |  |
| ***Age group*** | *1* | *0. 53* | *0.48* | *0.02* |
| *Residuals* | *28* |  |  |  |
| ***Condition*** | *1* | *1.06* | *0.31* | *0.04* |
| *Residuals* | *28* |  |  |  |
| ***Age group × Condition*** | *1* | *0.47* | *0.52* | *0.02* |
| *Residuals* | *28* |  |  |  |
| **Correct trials, only** | | | | |
| ***Age group*** | *1* | *1.92* | *0.18* | *0.06* |
| *Residuals* | *29* |  |  |  |
| ***Condition*** | *1* | *1.00* | *0.33* | *0.03* |
| *Residuals* | *29* |  |  |  |
| ***Age group × Condition*** | *1* | *1.48* | *0.23* | *0.05* |
| *Residuals* | *29* |  |  |  |

**Supplementary figure 1**


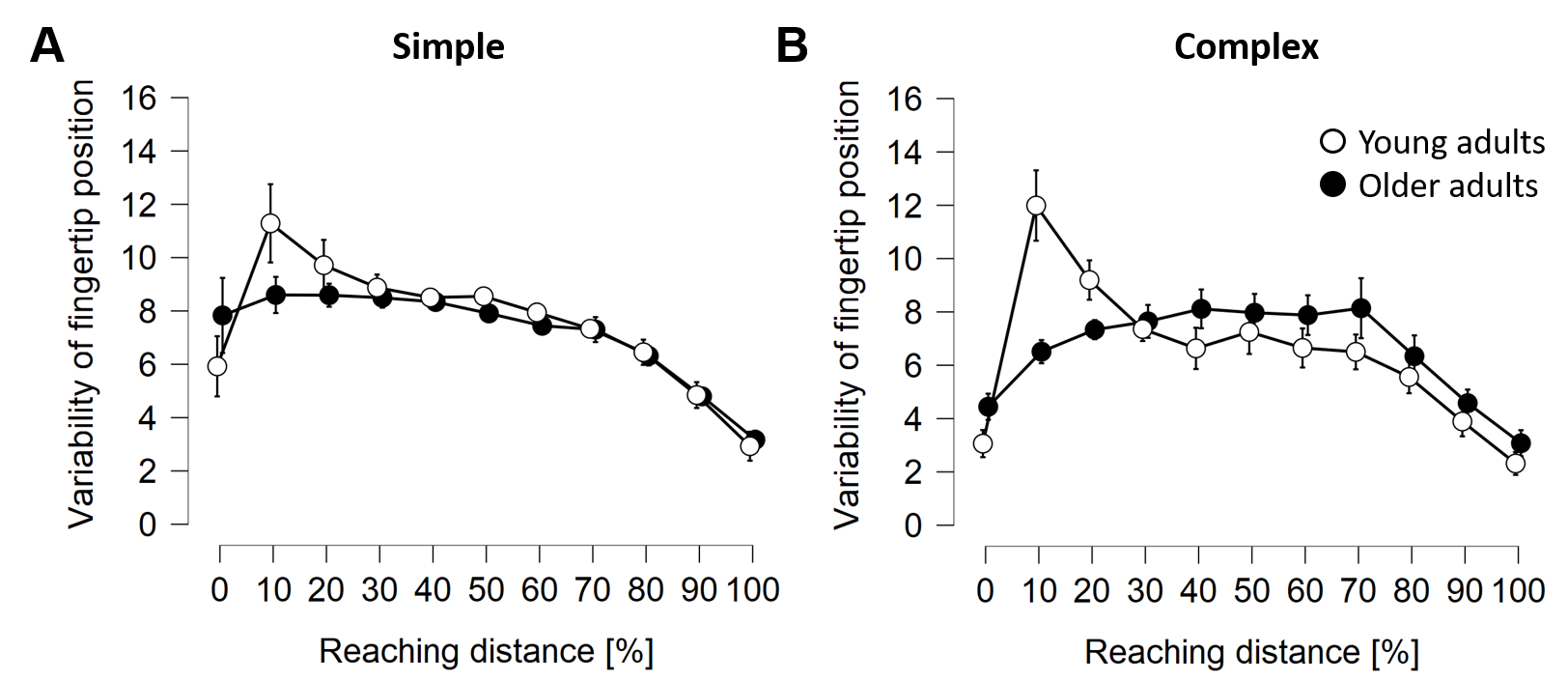


**Supplementary figure 1** – Depiction of the three-way interaction of Age group × Condition × Pointing sample for variability in fingertip position for correct trials only. Mean values ± SEM are depicted. **A** Time course of variability in fingertip position for young and older adults in the simple condition. **B** Time course of variability in fingertip position for young and older adults in the complex conditions.
